# The effect of chili pepper-Chinese chives intercropping on rhizosphere microorganisms and root-stem endophytes

**DOI:** 10.3389/fmicb.2025.1716326

**Published:** 2025-11-27

**Authors:** Nan Sun, Yuxin Wang, Ming Zhang, Peifang Ma, Zhao Wang, Haikang Zhao, Caixia Cao

**Affiliations:** 1 Department of Agricultural Structure and Environmental Engineering, College of Water Resources & Civil Engineering, China Agricultural University, Beijing, China; 2 Key Laboratory of Agricultural Engineering in Structure and Environment Ministry of Agriculture and Rural Affairs, Beijing, China; 3 Pingdingshan Academy of Agricultural Sciences, Pingdingshan, China; 4 Pingdingshan Pingfeng Seed Industry Limited Liability Company, Pingdingshan, China; 5 Beijing Agricultural Technology Extension Station, Beijing, China

**Keywords:** pepper-Chinese chives intercropping, microbial community reorganization, functional bacterial communities, co-occurrence network analysis, microecological regulation

## Abstract

**Introduction:**

This study adopted the intercropping pattern opepper (*Capsicum annuum* L.) and Chinese chives (*Allium tuberosum*), combined with high-throughput sequencing and microbial network analysis, to systematically reveal the mechanisms of intercropping on the structural regulation and functional synergy of the crop rhizosphere microbiome and root-stem endophyte communities.

**Methods:**

Three treatments were set up: blank control, solo cultivation, and intercropping.Combined with high-throughput sequencing and network analysis, the reorganization patterns of rhizosphere and endophyte communities were systematically analyzed.

**Results:**

Intercropping induced differential responses of microbial communities in the two crops: it significantly increased the bacterial α-diversity in Chinese chives leaves, and the Shannon index of pepper roots also showed an upward trend, while the microbial diversity in pepper rhizosphere soil was inhibited. In contrast, among roots, the “pepper intercropped with Chinese chives” group had the highest total number of OTUs and the largest number of unique OTUs. Microbial communities exhibited cross-host transfer characteristics: the migration rate of microbial communities from pepper roots to Chinese chives rhizosphere reached 46.57%, and 69.54% of the microbial communities in Chinese chives roots originated from pepper roots. Specifically, *Aureimonas* and Sphingomonadaceae were significantly enriched in pepper leaves, the relative abundance of *Pantoea* in Chinese chives leaves increased by 11.5 times, and the abundance of *Flavobacterium* in pepper rhizosphere increased by 94%. Microbial co-occurrence network analysis confirmed the optimization of functional synergy: the proportion of positive interactions in pepper leaves increased to 90.45%, and the negative interactions of *Bradyrhizobium* decreased by 97%, the proportion of positive interactions of functional bacteria in Chinese chives rhizosphere reached 88.96%, and *Bacillus* enhanced positive connections while maintaining an abundance of 10.23%–20.87%, the number of positive interactions of *Streptomyces* in pepper rhizosphere doubled. Network stability showed spatial variation: the robustness of stem microbial networks was significantly improved, while the vulnerability of rhizosphere microbial networks increased.

**Discussion:**

This study provides microbial theoretical support for the intercropping system to optimize nitrogen utilization by driving pepper to enrich the growth-promoting bacteria Sphingomonadaceae, and to enhance disease resistance by promoting Chinese chives to recruit the biocontrol bacteria *Bacillus*, thereby forming a microecological regulation mechanism with functional complementarity.

## 1 Introduction

Intercropping is an agricultural model that involves the communal cultivation of two or more crop species (Lithourgidis et al., 2011) in the same field. Compared with monocropping, intercropping makes significant contributions (Inal et al., 2007) to improving crop production efficiency and system stability by efficiently utilizing resources such as light, temperature, water and fertilizers.

In agricultural production systems, intercropping can significantly suppress soil-borne diseases and enhance crop health through mechanisms including diluting the density of pathogenic hosts, activating the secretion of antibacterial substances and recruiting beneficial rhizosphere microorganisms (Mommer et al., 2018; van Ruijven et al., 2020). Pepper is a globally important vegetable, but soil-borne diseases occur frequently. Chinese chives contain unique antibacterial metabolites and are preferred crops for intercropping to improve soil quality. However, the microbial interaction mechanism between the two in intercropping has not been clarified yet. Due to their antibacterial secondary metabolites, Allium plants are widely used in intercropping systems to control soil-borne pathogens (Abdel-Monaim and Abo-Elyousr, 2012; Hage-Ahmed et al., 2013). Intercropping of pepper (*Capsicum annuum* L.) with other crops is an effective and economical measure to restrict the cross-row spread of *Phytophthora capsici* in soil (Yang et al., 2014; Yang et al., 2022). Meanwhile, plant interspecific interactions are the core driving force shaping natural community structures and the functions of agricultural ecosystems. Their positive effects (e.g., promotion) can enhance the adaptability and resource utilization capacity of neighboring plants by improving the local microenvironment and regulating the composition of rhizosphere microbial communities (Brooker et al., 2021; Cardinale et al., 2012). Moreover, clarifying the key role of belowground interspecific interactions is crucial for regulating nitrogen (N) cycling in the plant–soil system and mediating the mineralization of soil organic matter (OM) (Chen et al., 2018; Guo et al., 2020; Zhao et al., 2022).

The rhizosphere, as a key zone for roots to acquire water and nutrients, has a close interaction between its microenvironment and soil physical structure, chemical properties as well as biological community composition (Inal et al., 2007). Plant roots provide the main nutrient source for soil microorganisms by releasing rhizosphere exudates (Pii et al., 2016), which in turn drives the dynamic changes in microbial community composition and metabolic activities. However, long-term continuous monocropping and irrational fertilization management have disrupted the balanced relationship among soil, microorganisms and plants, leading to the deterioration of soil physical and chemical properties and the imbalance of biological community structure (Inal et al., 2007; Zheng et al., 2021). It can be seen that the interaction process at the root-soil interface is a key link in maintaining soil health, ensuring sustainable food security and improving resource utilization efficiency (Wang X. et al., 2020). The optimization effect of intercropping on agricultural systems is also often achieved by regulating the rhizosphere microenvironment and root-soil interaction processes.

As an important component of agricultural ecosystems, the structure and function of soil microbial communities are comprehensively regulated by various factors such as soil physical and chemical properties, plant diversity and agricultural management practices (e.g., cultivation patterns) (Tian et al., 2019; Wu et al., 2012). Intercropping can improve soil nutrient cycling and enhance land productivity by regulating the composition and activity of microbial communities (Sun et al., 2022). In-depth studies have shown that constructing plant diversity can significantly enhance the disturbance resistance of ecosystems and agricultural sustainability by driving the functional reorganization of soil microbiomes (e.g., enriching beneficial microorganisms such as *Bacillus*, *Pseudomonas* and *Rhizobium*) (Ali et al., 2021; Ghani et al., 2022). These functional microorganisms can improve the soil microenvironment and nutrient availability by synthesizing plant hormones, secreting organic acids and fixing atmospheric nitrogen, thereby directly or indirectly promoting crop development (Hu et al., 2021; Lan et al., 2023; Wang et al., 2024). However, current relevant studies mostly focus on monoculture or traditional rotation patterns in field systems, and there are still relatively few studies on the effects of diversified planting systems (especially intercropping) on the structure and function of microbial communities in controlled environments. Current studies on intercropping of peppers mainly focus on yield and disease control, lacking systematic analysis of the recombination rules of rhizosphere-endophytic bacteria across ecological niches and the collaborative mechanisms of functional bacterial communities. Therefore, comprehensively exploring the regulatory effects of different planting systems on microbial community changes under protected conditions and their correlation mechanisms with agricultural sustainability and soil health has important theoretical and practical significance for optimizing crop management measures and improving agricultural productivity.

Based on the above context, this study proposes a hypothesis: The intercropping of Chinese chives and chili peppers can drive the directional enrichment and interaction of functional microbial communities, thereby mediating the reorganization of the microbial community, enhance the stability of functional bacterial interaction networks and thus optimize plant microecological health. To verify this hypothesis, this study combines high-throughput sequencing and network analysis to (1) analyze the effects of intercropping on the microbial composition of three niches (leaves, roots and rhizosphere) of Chinese chives and pepper (2) reveal the interaction patterns of key functional bacterial groups (e.g., growth-promoting bacteria, antibacterial genera) in the intercropping system (3) evaluate the correlation between microbial network robustness and disease suppression potential, so as to provide a theoretical basis for the design of intercropping systems.

## 2 Materials and methods

### 2.1 Sampling site and method

This study was conducted at the Changping Experimental Base of China Agricultural University (40°10’ N, 116°17′E). On February 22, 2023, samples were collected randomly according to the experimental design as follows: bulbs, roots and rhizosphere soil of solo-cultivated Chinese chives, solo-cultivated pepper, pepper intercropped with Chinese chives (<30 cm), Chinese chives intercropped with pepper (<30 cm), as well as plant-free control soil. Each treatment was set up with three biological replicates. Specifically, three adjacent plants were randomly selected from each treatment group for sampling, while for the control soil, three sampling points were randomly chosen within the corresponding treatment area for collection., resulting in a total of 39 samples. For rhizosphere soil sampling, healthy roots were dug out along the base of the plants, loose soil attached to the roots was shaken off, and the soil tightly bound to the roots (approximately 1 mm attached to the root system) was retained (Courchesne and Gobran, 1997). The samples were sealed in sterile plastic bags, stored in a car refrigerator, and transported to the laboratory.

### 2.2 Sample processing

In this experiment, rhizosphere soils from the same cultivation environment were mixed thoroughly. After removing visible soil animals and plant residues from the soil samples, they were passed through a 0.2 mm sieve. The samples were surface sterilized by washing with sterile water for 0.5 min, followed by washing in 75% ethanol for 1 min, then in 2% NaClO for 3 min, and then transferred to 75% sterile ethanol for 1 min. Finally, the plant tissues were washed with sterile water for 0.5 min (Zhu et al., 2020). Subsequently, the cleaned stems and roots of Chinese chives and pepper were placed into 50 mL Falcon centrifuge tubes containing 25 mL PBS buffer (PBS: 130 mM NaCl, 7 mM Na₂HPO₄, 3 mM NaH₂PO₄, pH 7.4), shaken and cleaned at 180 r/min for 15 min, with a total of 3 cleaning treatments. Then, the moisture of each sample was blotted dry with sterile filter paper, and impurities on the samples were removed completely with sterile tweezers. Finally, the stems and roots of Chinese chives and pepper, and the corresponding soil were stored at −80 °C for extracting genomic DNA from microorganisms in various plant niches and soil, and for high-throughput sequencing.

### 2.3 DNA extraction

First, each frozen sample was homogenized 4 times using an MP Fastprep-24 5G Biological Sample Homogenizer (MP Biomedicals, USA) at 7200 rpm, with each homogenization lasting 30 s. Then, plant tissues such as stems and roots of Chinese chives and peppers and rhizosphere soil (0.1–0.2 g) were weighed. DNA was extracted using the Fast DNA SPIN kit (MP Biomedicals, USA) in accordance with the standard process provided by the manufacturer. Finally, the quality and concentration of DNA were determined using a NanoDrop 2000 UV–Vis Spectrophotometer (Thermo Scientific, USA) and verified by 1% agarose gel electrophoresis. High-quality DNA templates were uniformly diluted to 10 ng/μL for subsequent PCR reactions targeting.

### 2.4 Nested PCR

AmplificationNested PCR was employed (Xia et al., 2018). In the first round, degenerate PCR primers 799F-1392R were used, with a Barcode added to the 5′ end to distinguish different samples. The 20 μL PCR amplification system consisted of 4 μL of 5 × Fast Pfu Buffer, 2 μL of 2.5 mmol/L dNTPs, 0.8 μL of forward primer (5 μmol/L), 0.8 μL of reverse primer (5 μmol/L), 0.4 μL of FastPfu DNA Polymerase, 0.2 μL of BSA, 10 ng of template DNA and ddH₂O added to a final volume of 20 μL. The PCR amplification program was carried out in a PCR instrument (ABI GeneAmp® 9,700, USA) as follows: pre-denaturation at 95 °C for 3 min 27 cycles of denaturation at 95 °C for 30 s, annealing at 55 °C for 30 s and extension at 72 °C for 45 s and a final extension at 72 °C for 10 min. In the second round, primers 799F-1193R were used (with a Barcode added to the 5′ end for sample differentiation) to amplify the V5–V7 variable regions of the 16S rRNA gene (Wang Y. et al., 2020). The PCR amplification system was 20 μL and the program was as follows: pre-denaturation at 95 °C for 3 min 13 cycles of denaturation at 95 °C for 30 s, annealing at 55 °C for 30 s and extension at 72 °C for 45 s a final extension at 72 °C for 10 min and subsequent preservation at 10 °C.

### 2.5 High-throughput sequencing

PCR was repeated 3 times for each sample, and the 3 PCR products were mixed. Then, 2% agarose gel electrophoresis was performed for detection. The target fragments of approximately 400 bp were subjected to gel extraction using the AxyPrep DNA Gel Extraction Kit (Axygen Biosciences, USA), eluted with Tris–HCl, and rechecked by 2% agarose gel electrophoresis. Subsequently, the PCR products were quantified using a Quantus™ Fluorometer (Promega, USA). Finally, libraries were constructed with the NEXTflex® Rapid DNA-Seq Kit, and the purified PCR products were paired-end sequenced (2 × 300 nt) on the Illumina MiSeq platform (Illumina, San Diego, USA).

### 2.6 Sequencing data

Processing the off-machine data were analyzed using fastp software and FLASH software (http://www.cbcb.umd.edu/software/flash, version 1.2.7). The processing steps were as follows: barcode sequences in the reads were removed, quality control and paired-end assembly of the sequencing reads were performed, samples were distinguished based on the removed barcode sequences, and then chimeras were identified and removed to obtain optimized sequences. Chimera identification was referenced to the SILVA database, which was then used as the reference sequence. For nucleotide sequences longer than 250 bp, operational taxonomic units (OTUs) were clustered with 97% similarity using UPARSE software (http://drive5.com/uparse/ version 7.1) for proportional analysis of bacterial communities in different niches among samples from different cultivation environments. Finally, sequence alignment was performed using the Silva (Release 138) 16S rRNA gene database with the Rdp Classifier algorithm^1^ and a confidence threshold of 70% to determine the taxonomic status of microorganisms corresponding to the sequences (Amato et al., 2013).

### 2.7 Statistical analysis

Kruskal-Wallis test was used for inter-group difference analysis, and Bonferroni correction was applied to the *p*-values with significant differences. Box plots were generated using Origin. ANOSIM was employed to analyze inter-group differences. The ggplot2 package in R software (version 3.3.1) was used to draw bar charts of the relative abundance of phyla and genera, with Kruskal-Wallis test and Wilcoxon rank-sum test (for two groups) applied. The *p*-values for significant differences among phyla were evaluated based on the community abundance data of quadrats, and false discovery rate (FDR) was used for correction. Data of each group were calculated and analyzed using the average values of intra-group samples, and visualized using STAMP software. For the analysis of biomarkers in different samples, the LEfSe method (Segata et al., 2011) was used to obtain LDA values online through the interactive webpage.^2^ Biomarkers with statistical significance (*p* < 0.05) could be used to demonstrate differences in bacterial communities among different ecological compartments. Based on the OTU table, R packages (WGCNA, psych, reshape2 and igraph) in R software were used to calculate the topological properties of microbial co-occurrence networks, and then Gephi (0.10.1) was used to visualize the network analysis results. The Robustness and maximum vulnerability index of microbial networks were calculated using the method described by Yuan et al. (2021), and implemented in R software.

## 3 Results and analysis

### 3.1 Changes in *α*-diversity of microbial communities

A total of 5,511,618 high-quality reads were obtained from the 39 samples with an average of 141,323 reads per sample.

**Figure 1:**
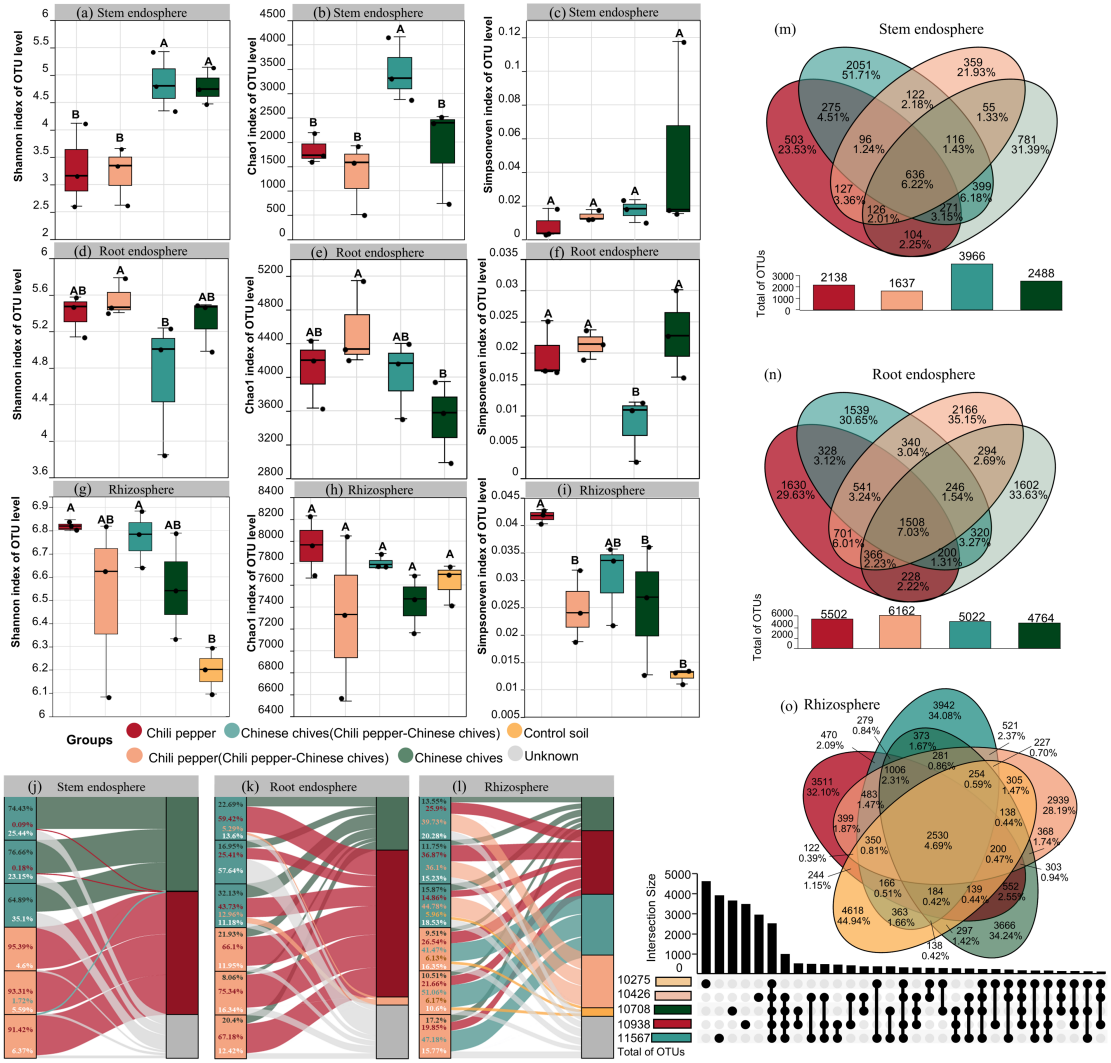
**(a–c)** Alpha diversity of the stem endosphere microbiome: **(a)** Shannon index, **(b)** Chao1 index, **(c)** Simpson index. **(d–f)** Alpha diversity of the root endosphere microbiome: **(d)** Shannon index, **(e)** Chao1 index, **(f)** Simpson index. **(g–i)** Alpha diversity of the rhizosphere microbiome: **(g)** Shannon index, **(h)** Chao1 index, **(i)** Simpson index; **(j–l)** Community composition of the microbiome (Sankey diagrams): **(j)** stem endosphere, **(k)** root endosphere, **(l)** rhizosphere. **(m–o)** Venn diagrams of operational taxonomic units (OTUs): **(m)** stem endosphere, **(n)** root endosphere, **(o)** rhizosphere. Different letters indicate significant differences among groups (*t*-test, *p* < 0.05). In the Venn diagrams, different colors represent different samples, the values in the overlapping areas indicate the number of OTUs shared by all samples, and the values in the non-overlapping areas indicate the number of unique OTUs for each sample.

Intercropping with pepper significantly increased the bacterial community diversity and species richness in Chinese chives leaves (*p* < 0.05, Figures 1a–c) but had no significant effect on the community diversity, species richness or community evenness in pepper leaves under intercropping (*p* > 0.05). Intercropping with Chinese chives increased the bacterial community diversity, species richness and community evenness in pepper roots yet the effect was not significant (*p* > 0.05, Figures 1d–f). The impact of intercropping on the community diversity of Chinese chives roots was greater than that on pepper roots and intercropping significantly decreased the Simpson evenness index of bacteria in Chinese chives roots (*p* < 0.05).

Regardless of intercropping, the community diversity and community evenness of rhizosphere soil in both Chinese chives and pepper were higher than those in the control soil, which was consistent with the trend of stem endophytes. Intercropping with pepper increased the bacterial Shannon index, Chao1 index and Simpson evenness index in Chinese chives rhizosphere soil (*p* > 0.05, Figures 1g–i). However, intercropping with Chinese chives reduced the bacterial Shannon index, Chao1 index and Simpson evenness index in pepper rhizosphere soil, weakening the original advantage of pepper rhizosphere soil (which had higher diversity indices than Chinese chives rhizosphere soil) and resulting in lower bacterial diversity and species richness in pepper rhizosphere soil than in Chinese chives rhizosphere soil.

### 3.2 Changes in *β*-diversity of microbial communities

There were significant differences in bacterial *β-*diversity. Among leaves, the “Chinese chives intercropped with pepper” group had the largest number of unique OTUs, accounting for 51.71% of all OTUs in leaves (Figure 1m). The largest number of shared OTUs between two groups was observed between solo-cultivated Chinese chives and “Chinese chives intercropped with pepper” with a total of 399. For pepper leaves under intercropping (i.e., “pepper intercropped with Chinese chives”), the total number of OTUs was the smallest (1637), which was much lower than that of “Chinese chives intercropped with pepper” (3966). However, this trend was completely opposite in roots: the “pepper intercropped with Chinese chives” group ranked first in the total number of OTUs (6162) and also had the largest number of unique OTUs (2166). In contrast, solo-cultivated pepper had the smallest number of unique OTUs, with only 1,630. Among the shared OTUs between two groups, the number of shared OTUs between solo-cultivated pepper and “pepper intercropped with Chinese chives” increased from 127 (in leaves) to 701 (in roots). Compared with leaves (636), the number of shared OTUs among the four groups (solo-cultivated pepper, “pepper intercropped with Chinese chives,” “Chinese chives intercropped with pepper” and solo-cultivated Chinese chives) increased to 1,508 in roots (Figure 1n).

In rhizosphere soil, the ranking trend of unique OTUs among the four groups was similar to that in leaves (Figure 1o). The “Chinese chives intercropped with pepper” group still had the largest number of unique OTUs (3942) while the “pepper intercropped with Chinese chives” group had the smallest (2939), yet both were lower than the number of unique OTUs in the control soil (4618). The number of shared OTUs between “pepper intercropped with Chinese chives” and “Chinese chives intercropped with pepper” was the largest among the three ecological compartments, reaching 521.

### 3.3 Analysis of microbial community origin

The origin of bacteria in Chinese chives leaves was relatively simple. The main origin of “Chinese chives intercropped with pepper” was Chinese chives leaves (average 71.99%) while the main origin of leaves of “pepper intercropped with Chinese chives” was pepper leaves (average 93.37%, Figure 1j).

Unexpectedly, the main origin of root bacteria in both “pepper intercropped with Chinese chives” (average 42.85%) and “Chinese chives intercropped with pepper” (average 69.54%) was pepper root bacteria followed by Chinese chives root bacteria (Figure 1k). A portion of root bacteria in “Chinese chives intercropped with pepper” originated from “pepper intercropped with Chinese chives” (average 6.08%) however no root bacteria in “pepper intercropped with Chinese chives” were found to originate from the roots of “Chinese chives intercropped with pepper.”

The main origin of bacteria in the rhizosphere soil of “Chinese chives intercropped with pepper” was the rhizosphere soil bacteria of “pepper intercropped with Chinese chives” with the highest source proportion reaching 44.78% followed by pepper rhizosphere soil bacteria (average 25.88%, Figure 1l). Only one rhizosphere soil sample of Chinese chives intercropped with pepper originated from control soil bacteria (5.96%). Similarly, the main origin of rhizosphere soil of “pepper intercropped with Chinese chives” was the rhizosphere soil of “Chinese chives intercropped with pepper” (average 46.57%) and the second source was also pepper rhizosphere soil bacteria (average 22.68%). The proportion of bacteria from unknown sources (average 14.24%) was lower than that in the rhizosphere soil of “Chinese chives intercropped with pepper” (average 18.01%).

### 3.4 Analysis of bacterial community structure

**Figure 2:**
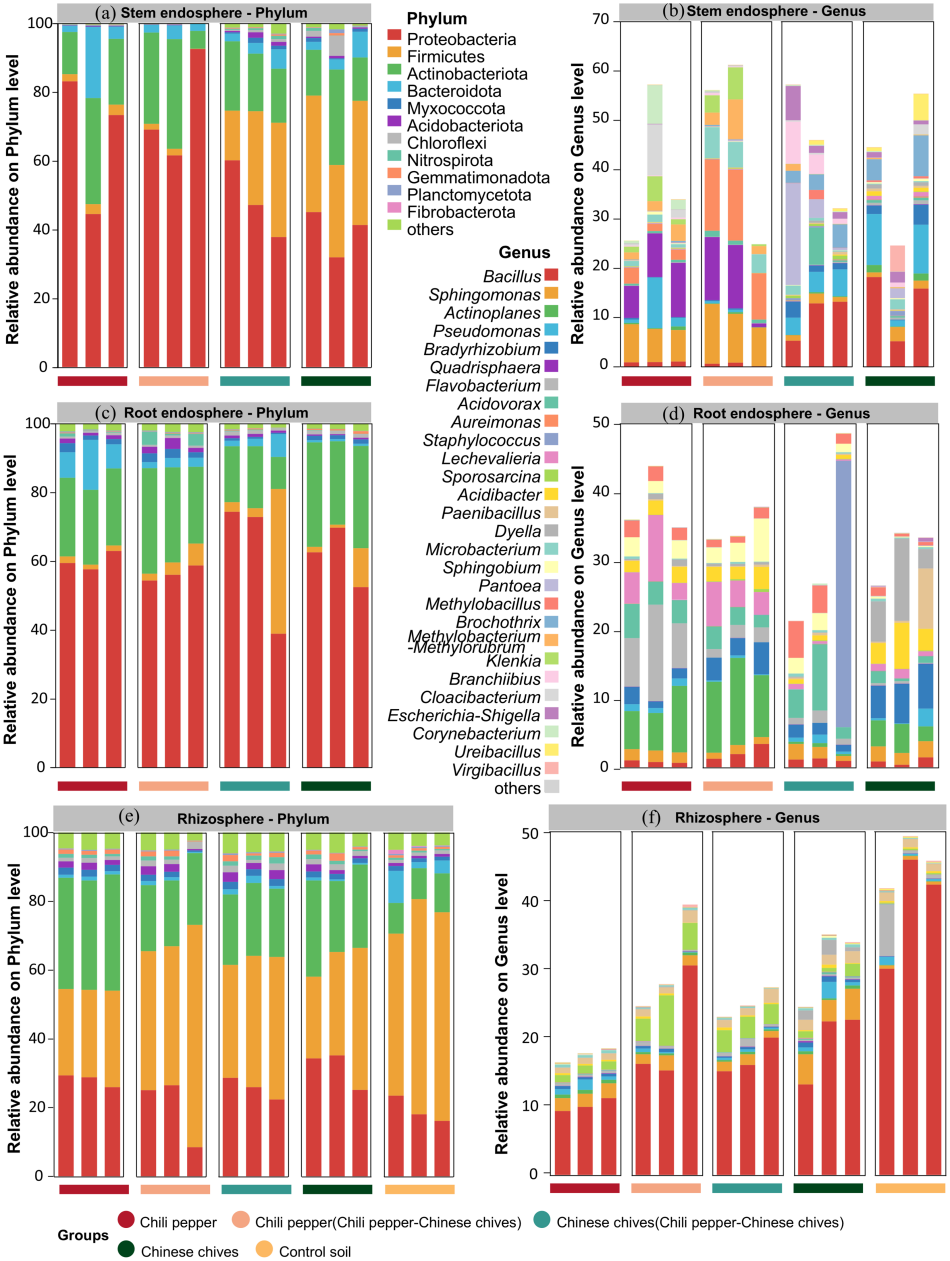
Relative abundance distribution of microbiome in different niches at phylum and genus levels: **(a)** Stem endosphere (phylum level), **(b)** Stem endosphere (genus level). **(c)** Root endosphere (phylum level), **(d)** Root endosphere (genus level). **(e)** Rhizosphere (phylum level), **(f)** Rhizosphere (genus level).

The phylum with the highest relative abundance of bacteria in leaves and roots was *Proteobacteria* while the one with the highest relative abundance in rhizosphere soil was *Firmicutes* (Figures 2a,c). At the genus level, the bacterial community structure of “Chinese chives intercropped with pepper” was relatively similar to that of solo-cultivated Chinese chives in leaves (Figure 2b). Both groups had *Bacillus* as the most abundant genus (10.36–12.99%) and *Pseudomonas* also ranked high in terms of relative abundance (4.36–7.14%). For *Pantoea*, its relative abundance was only 0.72% in solo-cultivated Chinese chives leaves but reached 8.33% in “Chinese chives intercropped with pepper” leaves making it the second most abundant known bacterial genus in “Chinese chives intercropped with pepper” leaves. The bacterial genera in “pepper intercropped with Chinese chives” leaves had low similarity to those in “Chinese chives intercropped with pepper” leaves yet showed a certain degree of similarity to those in solo-cultivated pepper leaves. For example, *Sphingomonas* was the second most abundant genus in both groups (6.94–9.98%). *Aureimonas* had the highest relative abundance (12.79%) in “pepper intercropped with Chinese chives” leaves while *Quadrisphaera* had the highest relative abundance (8.79%) in solo-cultivated pepper leaves.

In roots, *Flavobacterium* had the highest relative abundance (9.19%) in solo-cultivated pepper (Figure 2d) *Actinoplanes* had the highest relative abundance (10.69%) in “pepper intercropped with Chinese chives” *Staphylococcus* had the highest relative abundance (12.95%) in “Chinese chives intercropped with pepper” and *Dyella* had the highest relative abundance (6.89%) in solo-cultivated Chinese chives. Among the four groups, “pepper intercropped with Chinese chives” had the highest relative abundance of *Actinoplanes* (10.69%) followed by solo-cultivated pepper (6.90%) then solo-cultivated Chinese chives (3.39%) and finally “Chinese chives intercropped with pepper” (0.39%). The relative abundance of *Acidovorax* in “Chinese chives intercropped with pepper” roots (5.18%) was higher than that in “pepper intercropped with Chinese chives” roots (2.59%). After pepper was intercropped with Chinese chives, the relative abundance of *Bradyrhizobium* in its roots increased while the relative abundance of *Bradyrhizobium* in Chinese chives roots decreased after intercropping.

In rhizosphere soil, the five groups had the highest inter-group similarity at both the phylum and genus levels and the variation trends of various bacterial phyla and genera were very similar (Figures 2e,f). The three phyla with the highest relative abundance were all *Firmicutes* (23.81–64.66%) *Proteobacteria* (8.49–35.24%) and *Actinobacteriota* (8.89–33.76%). Among the four groups (solo-cultivated pepper, “pepper intercropped with Chinese chives,” “Chinese chives intercropped with pepper” and solo-cultivated Chinese chives) *Bacillus* was the bacterial genus with the highest relative abundance in rhizosphere soil (10.23–20.87%) and it also had the highest relative abundance in the control soil (39.86%). The relative abundance of *Sphingomonas* in the rhizosphere soil of solo-cultivated pepper and solo-cultivated Chinese chives was both higher than 2.0% but decreased to 1.69 and 1.32%, respectively, in the rhizosphere soil of “pepper intercropped with Chinese chives” and “Chinese chives intercropped with pepper.” *Pseudomonas* was another genus whose relative abundance decreased in the rhizosphere soil of intercropped plants. For instance, the relative abundance of *Pseudomonas* in solo-cultivated Chinese chives rhizosphere soil reached 1.16% even higher than that in the control soil (0.61%) but was only 0.30% in “Chinese chives intercropped with pepper” rhizosphere soil. In complete contrast to this trend was *Flavobacterium*: its relative abundance was only 0.35 and 0.23% in the rhizosphere soil of solo-cultivated pepper and solo-cultivated Chinese chives, respectively, but increased to 0.39 and 0.68% in the rhizosphere soil of “pepper intercropped with Chinese chives” and “Chinese chives intercropped with pepper” though both values were still lower than its relative abundance (2.89%) in the control soil.

### 3.5 Analysis of dominant bacterial communities in pepper and Chinese chives

**Figure 3:**
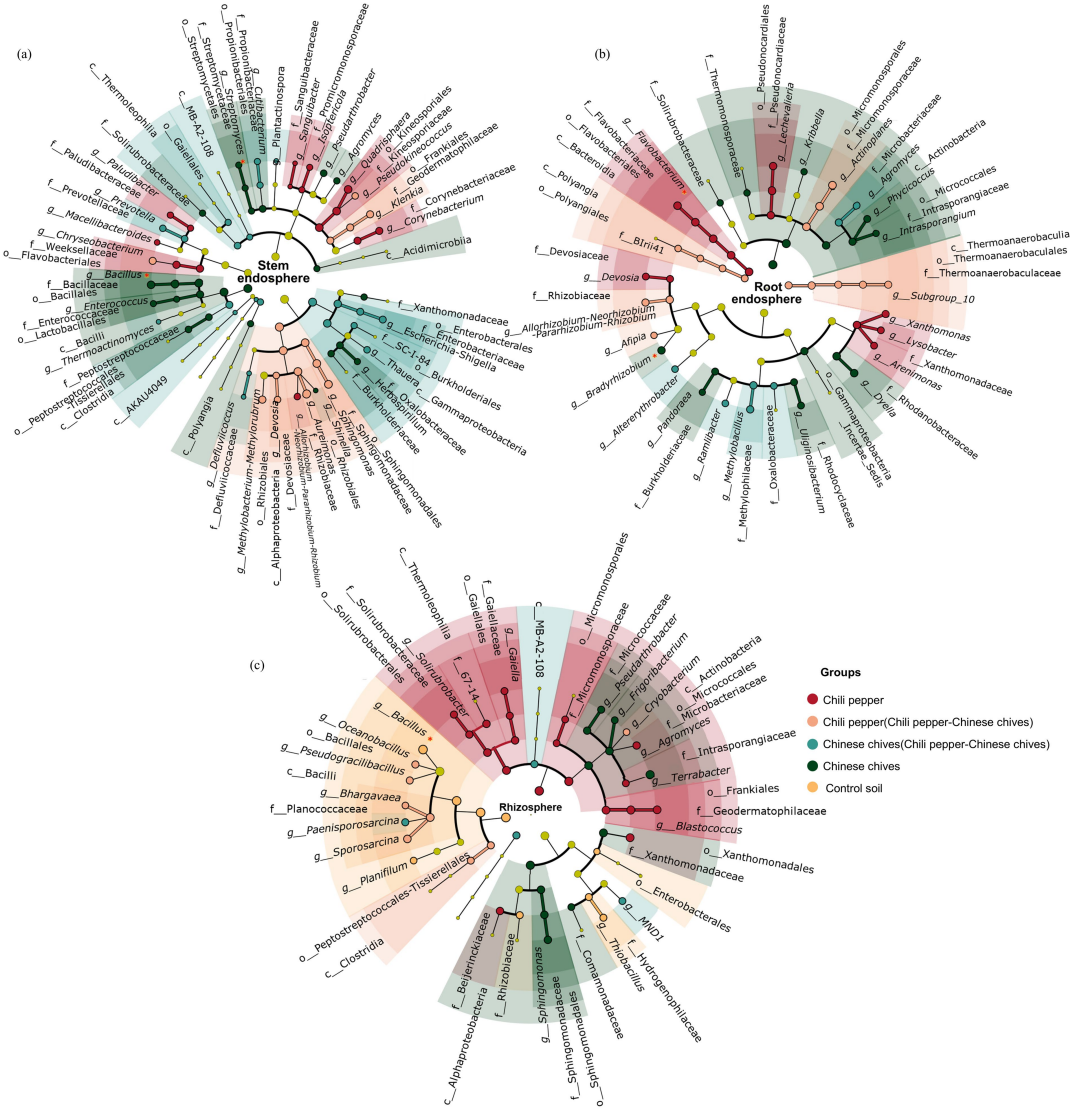
LEfSe analysis of microbiome in different niches in intercropping system (LDA > 2, *p* < 0.05): **(a)** Stem endosphere, **(b)** Root endosphere, **(c)** Rhizosphere; each ring from inside to outside represents taxonomic levels of phylum, class, order, family, genus in turn.

To further understand the impact of intercropping on changes in microbial communities of Chinese chives and pepper, this study conducted LDA analysis on dominant soil microbial communities, with emphasis on bacteria with broad-spectrum growth-promoting and antibacterial properties. At the genus level in leaves (Figure 3a), the top two with the highest LDA values were *Aureimonas* (LDA > 5.0) and *Sphingomonas* (LDA > 4.5), both significantly enriched in “pepper intercropped with Chinese chives.” The two families with the highest LDA values, *Rhizobiaceae* (LDA > 5.0) and *Sphingomonadaceae* (LDA > 4.5), were also significantly enriched in “pepper intercropped with Chinese chives.” *Quadrisphaera* (LDA > 4.5) and *Corynebacterium* (LDA > 4.0) were the two genera with the highest LDA values among those significantly enriched in solo-cultivated pepper, while *Kineosporiaceae* (LDA > 4.5) and *Weeksellaceae* (LDA > 4.0) were also significantly enriched in solo-cultivated pepper. Among the genera significantly enriched in “Chinese chives intercropped with pepper,” the ones with the highest LDA values were *Escherichia-Shigella* (LDA > 4.5) and *Defluviicoccus* (LDA > 3.5), and the families with relatively high LDA values at the family level were *Prevotellaceae* (LDA > 3.5) and *Propionibacteriaceae* (LDA > 3.5). The bacterial genera significantly enriched in solo-cultivated Chinese chives leaves were *Bacillus* (LDA > 4.5) and *Shinella* (LDA > 4.0), with *Bacillaceae* (LDA > 4.5) and *Oxalobacteraceae* (LDA > 4.0) significantly enriched at the family level with the highest LDA values.

In roots, the genus with the highest LDA value in solo-cultivated pepper was *Flavobacterium* (LDA > 4.5, Figure 3b) followed by *Lechevalieria* (LDA > 4.0), and the families with the highest LDA values at the family level were *Rhizobiaceae* (LDA > 4.0) and *Thermoanaerobaculaceae* (LDA > 4.0). In roots of “pepper intercropped with Chinese chives,” the genus with the highest LDA value at the genus level was *Actinoplanes* (LDA > 4.5) followed by *Allorhizobium-Neorhizobium-Pararhizobium-Rhizobium* (LDA > 4.0), and the families with the highest LDA values at the family level were *Micromonosporaceae* (LDA > 4.5) and *Rhizobiaceae* (LDA > 4.0). In roots of “Chinese chives intercropped with pepper,” the genus with the highest LDA value at the genus level was *Methylobacillus* (LDA > 4.5) followed by *Agromyces* (LDA > 4.0), and the families with the highest LDA values at the family level were *Methylophilaceae* (LDA > 4.0) and *Microbacteriaceae* (LDA > 4.0). In roots of solo-cultivated Chinese chives, the genus with the highest LDA value at the genus level was *Dyella* (LDA > 4.5) followed by *Bradyrhizobium* (LDA > 4.0), and the families with the highest LDA values at the family level were *Rhodanobacteraceae* (LDA > 4.0) and *Intrasporangiaceae* (LDA > 4.0).

In rhizosphere soil, the genus with the highest LDA value in solo-cultivated pepper was *Agromyces* (LDA > 4.0, Figure 3c) followed by *Blastococcus* (LDA > 4.0), and the families with the highest LDA values at the family level were 67–14 (LDA > 4.0) and *Beijerinckiaceae* (LDA > 4.0). In “pepper intercropped with Chinese chives,” the genus with the highest LDA value at the genus level was *Sporosarcina* (LDA > 4.0) followed by *Oceanobacillus* (LDA > 3.5). In “Chinese chives intercropped with pepper,” the genus with the highest LDA value at the genus level was *Paenisporosarcina* (LDA > 3.5) followed by MND1 (LDA > 3.5). In rhizosphere soil of solo-cultivated Chinese chives, the genus with the highest LDA value at the genus level was *Sphingomonas* (LDA > 4.0) followed by *Pseudarthrobacter* (LDA > 3.5), and the families with the highest LDA values at the family level were *Sphingomonadaceae* (LDA > 4.0) and *Microbacteriaceae* (LDA > 4.0).

### 3.6 Effects of intercropping on the complexity, stability, and vulnerability of bacterial interaction networks in pepper and Chinese chives

**Figure 4:**
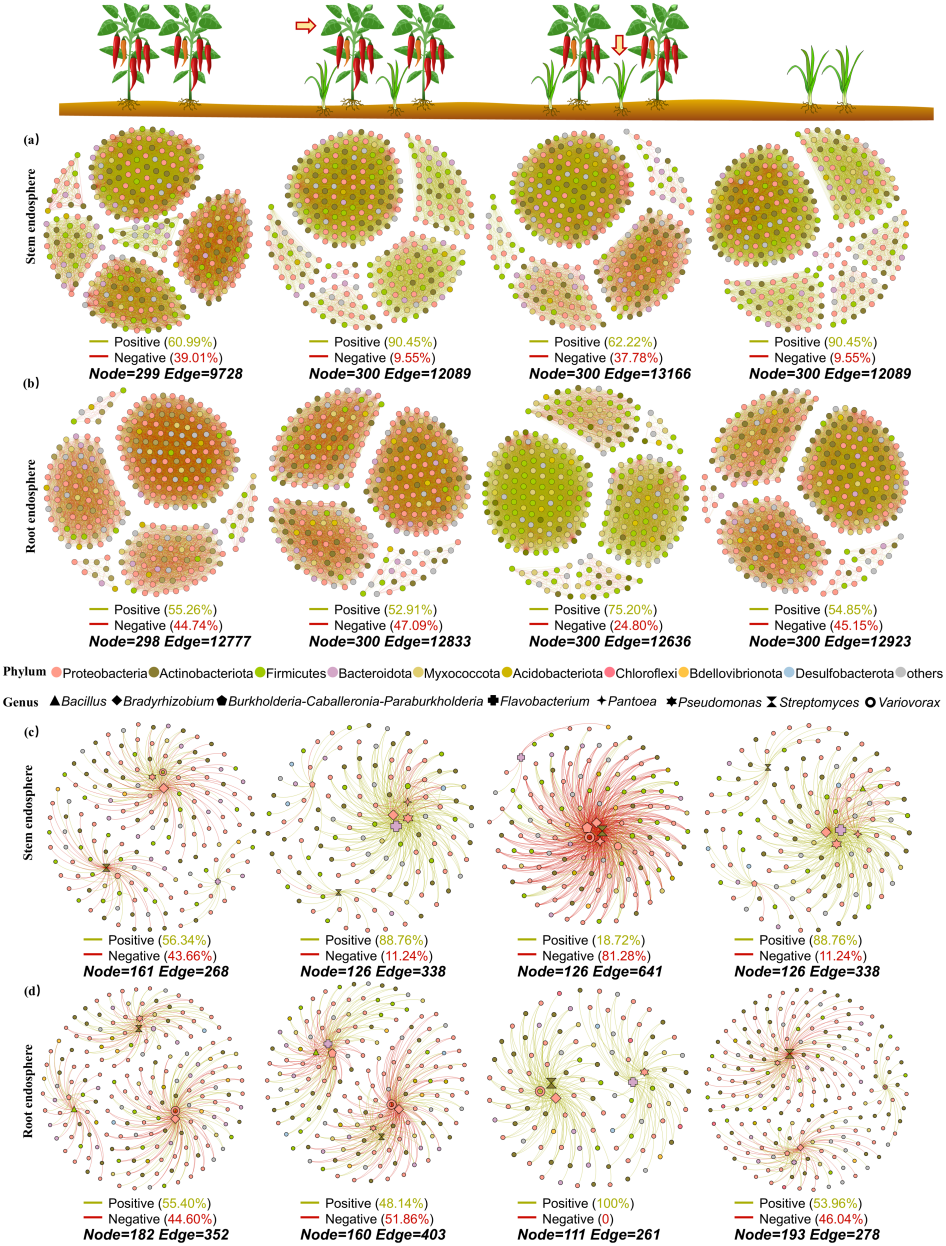
Co-occurrence network analysis of microbiome in different niches: **(a)** Stem endosphere, **(b)** Root endosphere. **(c)** Stem endosphere (selected functional bacterial genera), **(d)** Root endosphere (selected functional bacterial genera). among them, eight bacterial genera (*Bacillus*, *Bradyrhizobium*, *Burkholderia-Caballeronia-Paraburkholderia*, *Flavobacterium*, *Pantoea*, *Pseudomonas*, *Streptomyces*, and Var*iovorax*) related to plant growth promotion or resistance reported in Chinese chives or chili pepper were selected to construct the co-occurrence networks containing these functional bacteria before and after intercropping.

To compare the effects of intercropping on the complexity of bacterial co-occurrence networks in leaves, roots, and rhizosphere soil of pepper and Chinese chives, 300 nodes were selected from each group to construct 13 networks in this study. In leaves, the number of nodes in intercropped pepper (12089) and intercropped Chinese chives (13166) was higher than that in solo-cultivated pepper (9728) and solo-cultivated Chinese chives (12089) (Figure 4a). After pepper was intercropped with Chinese chives, the proportion of positive connections in the stem bacterial interaction network increased to 90.45% however after Chinese chives were intercropped with pepper, the proportion of negative connections in the stem bacterial interaction network increased from 9.55% (in solo cultivation) to 37.78%.

However, the trend of the root bacterial interaction network was the exact opposite (Figure 4b). After Chinese chives were intercropped with pepper, the proportion of positive interactions of bacteria in Chinese chives roots increased to 70.20% which was the highest proportion of positive connections in the root interaction networks among the four groups. The proportion of negative connections in pepper intercropped with Chinese chives increased by 2.35%. After pepper was intercropped with Chinese chives, the number of connections in the pepper root bacterial interaction network increased to 12,833 compared with solo-cultivated pepper which was higher than that in Chinese chives intercropped with pepper (12636).

**Figure 5:**
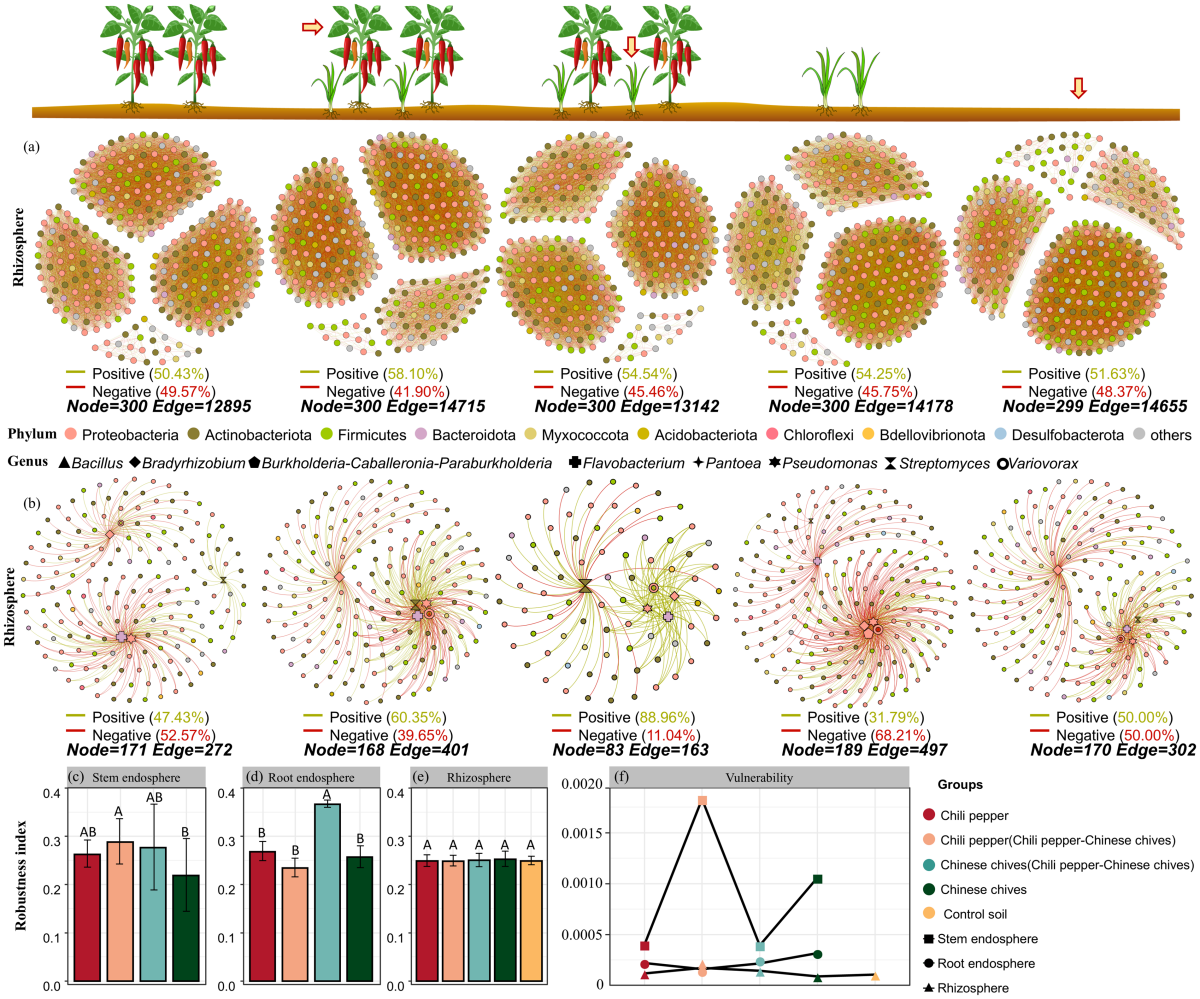
Co-occurrence network and analysis of robustness and vulnerability of microbiome in rhizosphere and different niches: **(a)** Co-occurrence network of rhizosphere (total microorganisms). **(b)** Co-occurrence network of rhizosphere (selected functional bacterial genera, eight bacterial genera *Bacillus*, *Bradyrhizobium*, *Burkholderia-Caballeronia-Paraburkholderia*, *Flavobacterium*, *Pantoea*, *Pseudomonas*, *Streptomyces*, and *Variovorax* related to plant growth promotion or resistance in Chinese chives or chili pepper were selected). **(c)** Intergroup differences of robustness index of co-occurrence network in stem endosphere. **(d)** Intergroup differences of robustness index of co-occurrence network in root endosphere. **(e)** Intergroup differences of robustness index of co-occurrence network in rhizosphere. **(f)** Network vulnerability of different groups.

In the rhizosphere soil bacterial interaction network, the number of connections in the solo-cultivated pepper bacterial interaction network was the lowest and the proportion of positive connections was also the lowest (50.43%) which was lower than that in the control soil (51.63%, Figure 5a). After pepper was intercropped with Chinese chives, it alleviated some negative connections in the interaction network and the proportion of positive connections increased to 58.10% which was the highest among all groups in rhizosphere soil. For the intercropped group of Chinese chives and pepper (13142) compared with solo-cultivated Chinese chives (14178), the number of connections in the bacterial interaction network decreased but the proportion of positive connections increased by 0.29% which was also higher than that in the control soil bacterial interaction network.

### 3.7 Robustness and vulnerability

To explore the effects of intercropping on the stability and vulnerability of bacterial co-occurrence networks in pepper and Chinese chives, we calculated network robustness by simulating species extinction. Based on random species loss (first, interaction networks containing all bacterial OTUs were constructed, and then 50% of the taxa were randomly removed from each network), it was found that in leaves, the robustness of the respective bacterial networks of pepper and Chinese chives increased after intercropping (Figure 5c). In particular, the robustness of the bacterial interaction network in “Chinese chives intercropped with pepper” leaves was significantly higher than that in solo-cultivated Chinese chives leaves (*p* < 0.05) and this trend was also verified in roots (Figure 5d).

After pepper was intercropped with Chinese chives, the robustness of the pepper stem bacterial network increased but not significantly (*p* > 0.05). In roots, its robustness was even lower than that of solo-cultivated pepper. No significant change in robustness was observed in rhizosphere soil (Figure 5e).

We also analyzed the maximum vulnerability of each network (Figure 5f) and found that the vulnerability of stem bacterial interaction networks was higher than that of other ecological compartments. In particular, the vulnerability of the bacterial interaction network in “pepper intercropped with Chinese chives” leaves was the highest, reaching 0.0018, followed by that in solo-cultivated Chinese chives leaves. Interestingly, in roots and rhizosphere soil, the changes in vulnerability of pepper and Chinese chives after intercropping showed completely opposite trends. In roots, the vulnerability of bacterial interaction networks of Chinese chives and pepper decreased after intercropping compared with that under solo cultivation while it increased in rhizosphere soil.

### 3.8 Effects of intercropping on interaction networks of growth-promoting and resistance-related bacterial genera

We screened bacterial genera previously reported to have growth-promoting or resistance-related properties in Chinese chives or pepper and finally identified eight bacterial genera: *Bacillus*, *Bradyrhizobium*, *Burkholderia-Caballeronia-Paraburkholderia*, *Flavobacterium*, *Pantoea*, *Pseudomonas*, *Streptomyces*, and Var*iovorax*. Bacterial interaction networks containing these functional bacteria before and after intercropping were constructed (Figures 4c,d, 5b). The bacterial interaction networks involving these functional bacteria in the leaves, roots, and rhizosphere soil of Chinese chives and pepper showed significant differences before and after intercropping.

In leaves, solo-cultivated pepper had the largest number of nodes of functional bacteria involved in the interaction network (161) but the smallest number of connections (268). After intercropping with Chinese chives, the proportion of positive connections in the pepper bacterial interaction network increased to 88.76%. *Bradyrhizobium* (−74) and *Streptomyces* (−43) exhibited numerous negative interactions with other bacterial genera in the leaves of solo-cultivated pepper but after intercropping with Chinese chives, the number of negative connections decreased to single digits (−7, −1) and the proportion of positive connections increased. This situation was completely opposite in Chinese chives leaves. Compared with solo-cultivated Chinese chives leaves (−7), the number of negative connections of *Bradyrhizobium* in “Chinese chives intercropped with pepper” leaves increased to −103. Additionally, the number of negative connections of Var*iovorax*, *Pantoea*, and *Burkholderia-Caballeronia-Paraburkholderia*—which are relatively active in Chinese chives—also increased after intercropping with pepper, resulting in an overall negative connection proportion of 81.28% in “Chinese chives intercropped with pepper” leaves. However, after intercropping with pepper, the number of positive connections of *Bacillus* increased to 8 and the number of negative connections decreased to 0.

In roots, the proportion of positive connections of pepper decreased to 48.14% after intercropping with Chinese chives while the proportion of positive connections of Chinese chives increased from 53.96 to 100% after intercropping. After intercropping with Chinese chives, the number of nodes of pepper decreased by 22 but the total number of connections increased to 402 making it the group with the largest number of connections in the root functional bacterial interaction network. Compared with solo-cultivated pepper, the number of negative connections of *Bacillus* in pepper decreased by 11 after intercropping with Chinese chives and its clustering distance with *Pantoea* and *Burkholderia-Caballeronia-Paraburkholderia* became shorter.

In the control soil of rhizosphere soil, the proportion of positive correlations and negative correlations in the functional bacterial interaction network was both 50%. After intercropping, the proportion of positive connections of functional bacteria in the rhizosphere soil increased for both Chinese chives (88.96%) and pepper (60.35%). Furthermore, the number of connections of pepper increased to 401 after intercropping with Chinese chives while the number of connections of Chinese chives decreased to 163 after intercropping with pepper. In pepper, the number of positive connections between *Streptomyces* and other bacterial genera increased from 22 to 47 after intercropping with Chinese chives and the number of positive interactions of *Bradyrhizobium* also increased by 30. Though the number of positive interactions of *Variovorax* increased from 22 to 48 its number of negative interactions also increased by 31. Intercropping with pepper decreased the number of connections of *Pseudomonas*, *Bradyrhizobium*, and *Flavobacterium* in Chinese chives especially the number of negative connections but significantly increased the number of positive connections of *Streptomyces*.

## 4 Discussions

This study revealed the reconstruction effect of the pepper-Chinese chives intercropping pattern on the rhizosphere microbiome and root-stem endophytic bacterial communities through multi-omics analysis and systematically elaborated for the first time the dynamics of bacterial interaction networks and the synergistic mechanism of functional bacterial communities in the intercropping system. The results showed that intercropping not only significantly changed the microbial community structure in different ecological compartments of the two crops but also reshaped the microbial ecological functions by regulating the interaction relationships of bacterial communities.

### 4.1 Functional bacterial communities’ directional changes

Intercropping induced distinct directional changes in functional bacterial communities across different ecological compartments of pepper and Chinese chives, with species-specific response patterns evident. For pepper, the bacterial diversity of its leaves was not significantly affected by intercropping (*p* < 0.05), but the relative abundance of Proteobacteria increased, and the proportion of positive interactions in the stem microbial co-occurrence network rose to 90.45%. Notably, the number of OTUs in the leaves of “pepper intercropped with Chinese chives” was only 41.3% of that in the leaves of “Chinese chives intercropped with pepper,” and the maximum vulnerability of the stem interaction network increased to the highest value among all interaction networks. Shifting to pepper roots, the increase range of the Shannon index of pepper roots after intercropping was larger than that of Chinese chives, suggesting that pepper roots may promote microbial colonization through more active metabolite exchange. Meanwhile, the relative abundances of *Bradyrhizobium* and *Bacillus* in the roots both increased, and the proportion of positive interactions of *Bacillus* also rose. Regarding pepper rhizosphere soil, the transfer intensity of pepper root microorganisms to Chinese chives rhizosphere was 46.57%, significantly higher than the reverse transfer (<6.08%), and *Aureimonas* was significantly enriched in the rhizosphere (LDA > 5.0). After intercropping, the number of positive interaction connections between *Streptomyces* and other bacteria in pepper rhizosphere increased significantly from 22 to 47, and its positive synergistic effect with *Bradyrhizobium* also increased significantly (by 30), which was consistent with the phenomenon that the total number of connections in the entire pepper rhizosphere functional bacterial network increased to 401. However, intercropping simultaneously inhibited the increase of microbial diversity in pepper rhizosphere soil, and both the vulnerability of the rhizosphere microbial interaction network and the maximum vulnerability of nodes increased. In the growth-promoting bacterial interaction network of pepper, *Bradyrhizobium*, *Streptomyces*, *Pseudomonas*, and *Flavobacterium* in its leaves and rhizosphere soil exhibited more interaction connections with other bacteria after intercropping. It is worth noting that when pepper was monocropped, there were a large number (74) of negative interactions between *Bradyrhizobium* and other bacteria in its leaves, but after intercropping, such negative interactions sharply decreased to only 7, and the proportion of positive interaction connections increased significantly.

In contrast to pepper, the response of functional bacterial communities in various organs of Chinese chives to intercropping showed unique characteristics. The bacterial diversity of Chinese chives leaves increased significantly after intercropping (*p* < 0.05). This phenomenon is not only related to the higher sensitivity of the phyllosphere microenvironment of Chinese chives to the volatile organic compounds (VOCs) of neighboring pepper but also can be explained by the characteristics of Amaryllidaceae plants—Chinese chives, as a plant of the same *Allium* genus as onions and garlic, can produce characteristic volatile substances (Greco et al., 2006; Hincapié and López, 2008), and its phyllosphere microenvironment may be more responsive to the chemical signals of neighboring crops due to its own metabolic characteristics, thereby driving differential changes in the bacterial community. Meanwhile, the proportion of negative interactions in the Chinese chives stem microbial network increased to 37.78%, and the number of OTUs in the leaves of “Chinese chives intercropped with pepper” was higher than that in the leaves of “pepper intercropped with Chinese chives.” The increase range of the Shannon index of Chinese chives roots was smaller than that of pepper, but after intercropping, although the number of connections in the Chinese chives root microbial interaction network decreased, the proportion of positive connections increased, and the overall robustness of Chinese chives improved, with the maximum vulnerability of root nodes decreasing. Similar to pepper, the relative abundance of *Bacillus* in Chinese chives roots and the proportion of its positive interactions also increased. At the rhizosphere soil level, intercropping promoted the increase of microbial diversity in Chinese chives rhizosphere soil. This result may be directly related to the interaction of physical and chemical properties of root exudates between the two crops, which is similar to the interspecific interaction mechanism in carrot-onion intercropping where onions exert an insect-repellent effect on carrot flies through chemical action (Uvah and Coaker, 1984). It is speculated that similar specific substance differences in root exudates between Chinese chives and pepper [such as the content of characteristic sulfur compounds in the essential oils of *Allium* plants like garlic, which is even 4 times higher than that in onions and broccoli (Attia et al., 2012)] may be the key driving the antagonistic changes of rhizosphere microbial diversity. In addition, the number of positive interaction connections of *Streptomyces* in Chinese chives rhizosphere surged, but the number of connections of *Pseudomonas* was inhibited, especially the number of negative interaction connections decreased. In terms of interaction network characteristics, the robustness of the Chinese chives stem interaction network increased significantly after intercropping (*p* < 0.05), and the maximum vulnerability of stem nodes also decreased.

### 4.2 Agronomic significance of microecological regulation

The microecological regulation induced by intercropping conferred substantial agronomic value, primarily manifested in optimized nitrogen utilization and enhanced disease prevention and control. From the perspective of nitrogen utilization, plants require nitrogen during growth, and previous studies have shown that a variety of nitrogen-fixing bacteria belong to Proteobacteria (Li et al., 2019; Rahav et al., 2016). Intercropping increased the relative abundance of Proteobacteria in both pepper and Chinese chives leaves and elevated the relative abundance of *Bradyrhizobium* in pepper roots, collectively facilitating the two crops’ acquisition of necessary nitrogen during the growth cycle. Additionally, rhizobia in the rhizosphere of pepper and Chinese chives provided more nitrogen for pepper through nitrogen fixation to meet its nitrogen demand, while root exudates of the two crops increased soil organic matter content and improved soil fertility. Actinobacteriota, which ranked third in relative abundance in the intercropping system, made significant contributions to nitrogen fixation (Nouioui et al., 2017), cellulose and lignin decomposition (Bull, 2011; Javed et al., 2021), and soil phosphate dissolution (Soumare et al., 2021), further optimizing nutrient cycling and utilization. Meanwhile, *Aureimonas* enriched in pepper rhizosphere has been proven to be widely involved in carbon and nitrogen cycles (Yoshida et al., 2019), enhancing pepper’s adaptability in nutrient competition.

Beyond nitrogen utilization optimization, the microecological regulation of intercropping also played a key role in disease prevention and control. *Bacillus* is widely distributed in agricultural soils and is associated with biological control of plant diseases, as well as plant growth promotion, quality improvement, and yield increase (Dame et al., 2021; Pérez-García et al., 2011). In facility greenhouses, *Bacillus* is often isolated from soil samples and root surfaces due to its acid resistance and endogenous spore-forming characteristics (Wu et al., 2025). After intercropping, the relative abundance of *Bacillus* in the roots of both pepper and Chinese chives increased, along with the proportion of its positive interactions. It is hypothesized that intercropping promotes *Bacillus* reproduction and that *Bacillus* is involved in organic matter decomposition, which is partially supported by Huang et al. (2022)—who used high-throughput sequencing to study the effect of intercropping on *Bacillus* species composition and found that *Bacillus amyloliquefaciens* isolated from intercropped soil exhibited excellent plant growth-promoting properties when co-inoculated with rhizobia—and Li et al. (2022)—who conducted high-throughput sequencing on soil microorganisms after intercropping apples with multiple plants and observed that *Bacillus* abundance in soil showed a synchronous increase with total carbon and available phosphorus, indicating a link between *Bacillus* proliferation and soil carbon-nitrogen cycles. Furthermore, Chinese chives enhanced antagonistic effects by increasing the proportion of negative interactions in their leaves, thereby inhibiting pathogen colonization. In the Chinese chives rhizosphere, the enhanced *Streptomyces* may partially replace or suppress *Pseudomonas* functionally, optimizing rhizosphere defensive capacity—and this is part of the “stress redistribution” and “functional complementarity” (Demie et al., 2022; Lang et al., 2022) of the two crops through regulating their associated microbial communities in the intercropping system. Notably, numerous studies have shown that intercropping can reduce pest issues (Li et al., 2020), and previous research has confirmed that intercropping regulates biological interactions via crop volatile substances [e.g., intercropping green beans with garlic decreases aphid numbers (Mohammadi et al., 2021)], providing a basis for pest prevention in the pepper-Chinese chives intercropping system.

### 4.3 Core ecological principles

Two core ecological principles underpin the microecological effects of the pepper-Chinese chives intercropping system: niche complementarity and microbial signal transduction. Niche complementarity is first reflected in underground niche differentiation: pepper is a deep-rooted crop, while Chinese chives is a shallow-rooted crop, and the two exhibit significant spatiotemporal niche dislocation. Chinese chives roots are mainly concentrated in the soil surface layer, while pepper roots can extend into the underground space of Chinese chives, thereby occupying resource advantages and providing conditions for increased microbial diversity in pepper roots—which aligns with previous research results (Zhang et al., 2021) that root distribution and rhizosphere effects influence interspecific interactions. In terms of resource allocation strategies, Chinese chives may prioritize resource allocation to above-ground parts in intercropping, while pepper focuses on underground microbial network construction, matching their root characteristics and achieving rational resource allocation.

Above-ground niche complementarity is equally significant: when intercropping plants with different canopy heights, taller canopy plants can obtain greater light interception and root extension, thereby gaining a competitive advantage (Gao et al., 2010). Pepper, with a taller canopy, acquires more light interception, while Chinese chives—as a low-stature crop—experiences partial inhibition in light resource acquisition after being shaded by pepper, which alters light resource utilization and affects the microbial diversity of Chinese chives leaves. Although intercropping improves overall photosynthetic utilization efficiency, the differentiated utilization of light resources achieves above-ground niche complementarity. Additionally, the environmental conditions of the intercropping system can affect interspecific competition (Zhang et al., 2015), and niche complementarity between pepper and Chinese chives alleviates interspecific competition to a certain extent, promoting the stable coexistence of the two crops and the optimization of microbial communities. Numerous studies have also shown that intercropping promotes biodiversity (Mahmoud et al., 2022), and the niche complementarity between pepper and Chinese chives provides a favorable environment for the survival and reproduction of different microorganisms, thereby enhancing microbial biodiversity.

Microbial signal transduction is another core principle by which intercropping regulates microbial community structure and function. Plant volatile substances play a central role in interspecific interactions: the phyllosphere microenvironment of Chinese chives is more sensitive to the VOCs of neighboring pepper, driving differential changes in the microbial community of Chinese chives leaves. As an *Allium* genus plant, Chinese chives can produce characteristic volatile substances (Greco et al., 2006; Hincapié and López, 2008), and its phyllosphere microenvironment may be more responsive to chemical signals from neighboring crops due to its own metabolic characteristics, further confirming the role of VOCs in microbial signal transduction. The physical and chemical properties of crop root exudates also profoundly affect microbial colonization: just as various substances released by *Allium* leaves can act as toxins, feeding deterrents, or insect repellents (Kant et al., 2009), the interaction of physical and chemical properties of root exudates between pepper and Chinese chives drives antagonistic changes in rhizosphere microbial diversity, and the more active metabolic exchange of pepper roots may give them an initiative in interspecific root chemical interactions.

Qiao et al.’s (2024) study on legume-non-legume interactions confirmed that non-legume companion crops (e.g., maize) can significantly enhance the nodulation efficiency and nitrogen-fixing activity of rhizosphere *Bradyrhizobium* by inducing the secretion of secondary metabolites such as flavonoids from legume (peanut) roots. Drawing on this mechanism, Chinese chives—as an *Allium* plant rich in flavonoids (Marefati et al., 2021)—may directly provide signal molecules such as flavonoids to stimulate the proliferation of *Bradyrhizobium* in the pepper rhizosphere when intercropped with pepper, and optimize the rhizosphere microenvironment while alleviating resource competition between *Bradyrhizobium* and other microorganisms through metabolite-mediated microbial interactions, thereby enhancing its colonization advantage in pepper roots. Interspecific interactions between crops can also regulate the interaction signals of microbial communities: for example, after intercropping, the co-occurrence network of pepper rhizosphere microorganisms has more connections and a higher proportion of positive connections under the same number of nodes—consistent with previous studies showing that intercropping increases the nodes, edges, and modularity of crop rhizosphere bacterial networks (Li and Wu, 2018; Pang et al., 2021)—and the differentiated “competition-cooperation” strategies of pepper and Chinese chives microbial networks (pepper enhances competitive advantage in nutrient acquisition by strengthening bacterial community cooperation, while Chinese chives inhibit pathogen colonization by increasing antagonistic effects) also reflect the regulation of microbial interaction signals.

Furthermore, interspecific synergy in intercropping can improve the soil environment, affect the survival of soil microorganisms, stimulate the activity of microorganisms related to nutrient transformation (e.g., carbon and nitrogen), enhance the absorption and utilization of soil nutrients by crops, and promote the rhizosphere to better recruit beneficial microorganisms (Gu et al., 2025)—a process accompanied by microbial signal transmission that promotes the optimization of microbial community structure and function. High-throughput sequencing data showed that Proteobacteria, Firmicutes, Actinobacteriota, and Bacteroidota were the top four phyla in the intercropping system, consistent with previous research results (Li et al., 2024; Niu et al., 2022); this consistency in dominant phyla reflects the regularity of microbial signal transmission in intercropping systems and provides a stable microbial basis for the functional exertion of the intercropping system.

The robustness index of interaction networks is evaluated by simulating random node removal (i.e., species extinction) and observing the rate of network structure collapse; a stable network can maintain most of its structure after species loss, demonstrating its ability to maintain structure and function under disturbances or attacks (Yuan et al., 2021). After intercropping, the robustness of the stem interaction networks of both crops increased significantly (*p* < 0.05), while the vulnerability of the rhizosphere networks increased—this may be because the above-ground microbial community developed stronger disturbance resistance in intercropping, while the underground community became more sensitive due to enhanced interspecific interactions, which is also confirmed by Dang et al. (2024), who showed that the robustness of the underground interaction network of soybeans decreased after intercropping. High vulnerability means that key nodes have a greater impact, the interaction network is more dependent on key nodes, and is prone to imbalance due to node changes; nodes with high maximum vulnerability are usually key nodes in the network, and their removal can lead to a significant decline in network efficiency or even local/global structural changes [e.g., in a microbial network, the loss of key species may disrupt ecological balance and cause community structure instability (Yuan et al., 2021)], reflecting the feedback effect of microbial network signals on the stability of the intercropping system.

Key taxa in microbial interaction networks usually have high relative abundance and fewer core OTUs, which may significantly affect network stability (Chen and Wen, 2021). Interspecific interactions between peanut and sorghum, as well as between wheat and broad bean, also help maintain the stability and ecological functions of microbial communities by reorganizing the originally stable core microbiome; compared with monoculture, the proportion of negative connections between fungi and bacteria in mixed intercropping decreases (Granzow et al., 2017; Shi et al., 2021), which also applies to the pepper-Chinese chives intercropping system, reflecting the reorganization of core microbiome signals driven by intercropping.

## 5 Conclusion

The pepper-Chinese chives intercropping system significantly optimizes the microecological environment of the two crops by driving microbial community reorganization and functional synergy. The study confirms that intercropping induces differentiated responses in the microbial communities of the two crops: the bacterial *α*-diversity and proportion of unique OTUs in Chinese chives leaves are significantly increased while the total number of OTUs and the enrichment degree of unique bacterial communities in pepper roots are the highest yet the rhizosphere diversity is inhibited.

Microorganisms exhibit cross-host migration characteristics the migration rate of pepper root microbial communities to the Chinese chives rhizosphere reaches 46.57 and 69.54% of the microbial communities in Chinese chives roots are derived from pepper accompanied by specific enrichment of functional bacteria (e.g., the abundance of *Aureimonas* in pepper leaves and *Pantoea* in Chinese chives leaves increased by 11.5 times).

Interaction network analysis reveals the functional synergy mechanism: the proportion of positive interactions in pepper leaves increases to 90.45% and the negative interactions of *Bradyrhizobium* decrease by 97% *Bacillus* in the Chinese chives rhizosphere enhances positive connections while maintaining high abundance.

This system optimizes nitrogen utilization by enriching growth-promoting bacteria (such as *Sphingomonadaceae*) in pepper and enhances disease resistance by recruiting biocontrol bacteria (such as *Bacillus*) in Chinese chives forming a microecological regulation model of “stress redistribution-functional complementarity” which provides a theoretical basis for the design of sustainable intercropping systems.

## Data Availability

The datasets presented in this study can be found in online repositories. The names of the repository/repositories and accession number(s) can be found below: https://www.ncbi.nlm.nih.gov/, PRJNA1336478.

## Footnotes

1
http://rdp.cme.msu.edu

2
http://huttenhower.sph.harvard.edu/galaxy/
